# Supplementary material for: International Music Therapists’ Perceptions and Experiences in Telehealth Music Therapy Provision
Source: Int J Environ Res Public Health. 2023 Apr 19;20(8):5580. doi: 10.3390/ijerph20085580 (PMC10139124; doi:10.3390/ijerph20085580)
Supplement: Supplementary file 1 [file ijerph-20-05580-s001.zip › Supplementary Material 1 _ Complete Survey.pdf]

Supplementary Material 1: Complete Survey

Music Therapists' Experiences in Telehealth Music Therapy Provision

For the purposes of this survey, telehealth music therapy (TMT) will refer to the delivery of music therapy services remotely using online platforms. TMT refers to a combination of synchronous audio and videoconferencing music therapy sessions and asynchronous approaches such as original recordings and instructions, web-based resources, and supportive directives for clients/patients and caregivers. This survey should take approximately 10-15 minutes to complete. We thank you in advance for your participation.

Do you currently provide telehealth music therapy (TMT) services? (If yes, proceed, if no, end survey)

1. Demographics

1. **Age** {Dropdown menu}
   Check this box if you prefer not to specify

2. **Gender**
   Female
   Male
   Non-binary/Non-conforming
   Prefer not to say
   Prefer to self-describe _______

3. **In which country do you currently live and practice music therapy?** {Dropdown}

4. **How many years have you been practicing as a music therapist?**
   Less than 5 years | 5-10 years | 10-15 | 15-20 | More than 20 years

5. **What is the highest education level you have completed?**
   Bachelor's Degree
   Master's Degree
   Doctorate Degree
   Graduate Certificate
   Other (please specify) _______

2. Clinical Practice

6. **What primary theoretical orientation do you utilize when providing TMT?** {Select one}

   Neurologic Music Therapy

   Nordoff-Robbins Music Therapy

   The Bonny Method of Guided Imagery and Music

   Music Psychotherapy

   Humanistic

   Eclectic and /or Integrative

   Aesthetic Music Therapy

   Other (please specify) __________

7. **What other theoretical orientations do you utilize when providing TMT? {Select the next two most prominent}**

   Neurologic Music Therapy

   Nordoff-Robbins Music Therapy

   The Bonny Method of Guided Imagery and Music

   Music Psychotherapy

   Humanistic

   Eclectic and /or Integrative

   Aesthetic Music Therapy

   Other (please specify) ____________

8. **What is your current primary work setting?**

   Currently Unemployed

   Private Practice

   Academia- Primary/Secondary Education

   Academia- Tertiary/Higher Education

   Healthcare/Medical facility (e.g., hospital, rehabilitation center, long-term care facility)

   Correctional Facility

   Military or Veteran Medical Center

   Specialized Clinic (e.g., pain, oncology, brain injury)

Community Music/Arts Center
Other (please specify) ____________

9. Prior to COVID-19, which clinical populations did you primarily serve?
{Select up to three}

Children with developmental delays
Persons with Intellectual/Multiples Disabilities
Children/Youth at risk
Adolescents
Older Adults
Palliative Care/End of Life
Dementia
Medical/Oncology
Premature Infants
Mental Health
Neurorehabilitation (Stroke/Traumatic Brain Injury)
Other (please specify) ________

10. Since COVID-19, which clinical populations do you primarily serve?
{Select up to three}

Children with developmental delays
Persons with Intellectual/Multiples Disabilities
Children/Youth at risk
Adolescents
Older Adults
Palliative Care/End of Life
Dementia
Medical/Oncology
Premature Infants
Mental Health
Neurorehabilitation (Stroke/Traumatic Brain Injury)
Other (please specify) ________

11. How many clinical hours per week did you have <u>prior</u> to COVID-19?

Not applicable (instructor/administrator)

1-9 hours per week
10-19 hours per week
20-29 hours per week
30-35 hours per week
36 hours or more per week

12. How many clinical hours per week have you had since COVID-19?
Not applicable (instructor/administrator)
1-9 hours per week
10-19 hours per week
20-29 hours per week
30-35 hours per week
36 hours or more per week

3. Telehealth Provision

13. What terminology do you most commonly use to describe your non-in-person MT services? {select one}
Online Music Therapy
Remote Music Therapy
Telehealth Music Therapy
Virtual Music Therapy
Distance Delivery of Music Therapy
Other (please specify) ________

14. Have you been providing TMT prior to the pandemic? {For more than 5 sessions}
Yes, No

15. What clinical needs do you address with TMT? {Select top three}
Pain
Mood
Anxiety
Isolation
Speech and Language / Communication
Cognition

Motor Function

Spiritual Support

Emotional Expression

Other (please specify) ________

16. **Have your client's goal areas changed due to the transition to telehealth compared to in-person sessions?**

    {Not at all - For some clients - Unsure - Likely - Yes, definitely}

17. **What are the most frequently used interventions during your TMT?** {Select top two}

    Music Listening

    Improvisation

    Songwriting

    Singing

    Mindfulness

    Movement to Music

    Other (please specify) ________

18. **I have successfully used <u>live music</u> during:**

    In-person MT: Strongly agree - Agree - Neither agree nor disagree - Disagree - Strongly disagree

    Telehealth MT: Strongly agree - Agree - Neither agree nor disagree - Disagree - Strongly disagree

19. **I have successfully used <u>pre-recorded music</u> during:**

    In-person MT: Strongly agree - Agree - Neither agree nor disagree - Disagree - Strongly disagree

    Telehealth MT: Strongly agree - Agree - Neither agree nor disagree - Disagree - Strongly disagree

20. **What outcome measures do you use to assess progress in telehealth MT?**

    Client Self-Report (e.g., Likert scales, verbal, written)

    Family/Caregiver Report (e.g., interviews, session feedback, verbal, written)

    Observation (e.g., therapist, client behavior)

    Standardized Assessments

Diagnostic assessments performed by other healthcare professionals

21. In general, I am able to administer assessments over telehealth:

{Strongly agree - Agree - Neither agree nor disagree - Disagree - Strongly disagree}

22. What online platform did you primarily use to administer TMT?

Zoom

Skype

Teams

Adobe Connect

Facetime

Google Duo

Other (please specify) ________

23. Did these platform(s) comply with legal or confidentiality requirements (e.g., HIPAA) in your country?

Yes
No
Not sure

24. Did you take training, workshops, and/or courses to develop skills or increase proficiency of TMT service provision?

Yes
No
If yes, please specify:

4. Telehealth Perceptions

25. Do you anticipate continuing to provide TMT services post-pandemic?

{Not at all - For some clients - Unsure - Likely - Yes, definitely}

26. Do you anticipate that the frequency of your telehealth services will decline when restrictions for COVID-19 are removed completely?

Yes | No

26b. If yes, by what percentage (projected):

10 – 25 – 50 – 75

27. TMT has more benefits than drawbacks:

{Strongly agree - Agree - Neither agree nor disagree - Disagree - Strongly disagree}

28. Caregiver involvement is beneficial for a telehealth model:

{Strongly agree - Agree - Neither agree nor disagree - Disagree - Strongly disagree}

29. Please list the main challenges for your clients in engaging in a telehealth MT session:

30. Please list the main challenges for yourself in engaging in a telehealth MT session:

31. Please list the main benefits for your clients in engaging in a telehealth MT session:

32. Please list the main benefits to you from engaging in a telehealth MT session:

33. If you are an MT supervisor, please describe challenges with:

a. Providing supervision in TMT

b. Supervisee skill acquisition in TMT
