# Supplementary material for: International Music Therapists’ Perceptions and Experiences in Telehealth Music Therapy Provision
Source: Int J Environ Res Public Health. 2023 Apr 19;20(8):5580. doi: 10.3390/ijerph20085580 (PMC10139124; doi:10.3390/ijerph20085580)
Supplement: Supplementary file 1 [file ijerph-20-05580-s001.zip › Supplementary Material 2 _ Survey Responses (1).pdf]

Supplementary Material 2. Survey Responses

Music Therapists' Experiences in Telehealth Music Therapy Provision

1. Demographics

1. Age

Age

N	Valid	563
	Missing	9
Mean		41.29
Median		39.00
Std. Deviation		12.812
Range		62
Minimum		23
Maximum		85

2. Gender

Gender

	N	%
Female	473	82.7%
Male	80	14.0%
Non-binary/Non-conforming	10	1.7%
Prefer not to say	7	1.2%
Other	2	0.3%
Total	572	100%

3. In which country do you currently live and practice music therapy?

Country

	N	%
United States	386	67.5%
Canada	60	10.5%
Australia	52	9.1%
Italy	12	2.1%
Ireland	8	1.4%
Thailand	6	1.0%

China	5	0.9%
Malaysia	5	0.9%
New Zealand	5	0.9%
United Kingdom	5	0.9%
Brazil	4	0.7%
Argentina	2	0.3%
Chile	2	0.3%
Colombia	2	0.3%
Israel	2	0.3%
Singapore	2	0.3%
Taiwan	2	0.3%
Croatia	1	0.2%
Denmark	1	0.2%
Finland	1	0.2%
Germany	1	0.2%
India	1	0.2%
Lebanon	1	0.2%
Luxembourg	1	0.2%
Philippines	1	0.2%
Poland	1	0.2%
Portugal	1	0.2%
Spain	1	0.2%
Turkey	1	0.2%

Country - per region

	N	%
North America	446	78.0%
South America	10	1.7%
Asia	26	4.5%
Oceania	57	10.0%
Europa	33	5.8%
Total	572	100%

4. How many years have you been practicing as a music therapist?

Years practicing as a MT

	N	%
Less than 5 years	146	25.5%

5-10 years	152	26.6%
10-15 years	93	16.3%
15-20 years	52	9.1%
More than 20 years	129	22.6%
Total	572	100%

5. What is the highest education level you have completed?

Education Level

		N	%
Bachelor's Degree		186	32.5%
Master's Degree		307	53.7%
Doctorate Degree		54	9.4%
Graduate Certificate		12	2.1%
Other		12	2.1%
Total		571	99.8%
Missing	System	1	0.2%

2. Clinical Practice

6. What primary theoretical orientation do you utilize when providing TMT?

Primary Theoretical Orientation for TMT

	N	%
Neurologic Music Therapy	50	8.7%
Nordoff-Robbins Music Therapy	27	4.7%
The Bonny Method of Guided Imagery and Music	14	2.4%
Music Psychotherapy	45	7.9%
Humanistic	177	30.9%
Eclectic and / or Integrative	200	35.0%
Aesthetic Music Therapy	1	0.2%
Other	58	10.1%
Total	572	100%

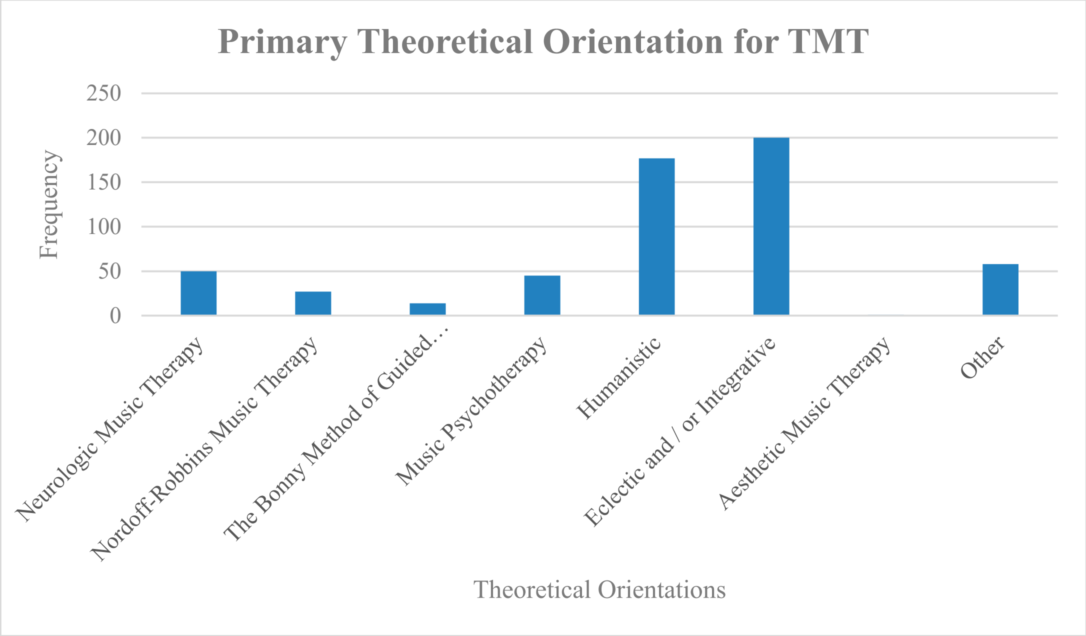

7. What other theoretical orientations do you utilize when providing TMT? {Select the next two most prominent}

Theoretical Orientations (2nd and 3rd options combined)

		Responses		Percent of Cases
		N	Percent	
TO23[a]	Neurologic Music Therapy	155	13.6%	27.1%
	Nordoff-Robbins Music Therapy	93	8.1%	16.3%
	The Bonny Method of Guided Imagery and Music	22	1.9%	3.8%
	Music Psychotherapy	155	13.6%	27.1%
	Humanistic	301	26.4%	52.6%
	Eclectic and / or Integrative	264	23.1%	46.2%
	Aesthetic Music Therapy	46	4.0%	8.0%
	Other	106	9.3%	18.5%
Total		1142	100.0%	199.7%

a. Group

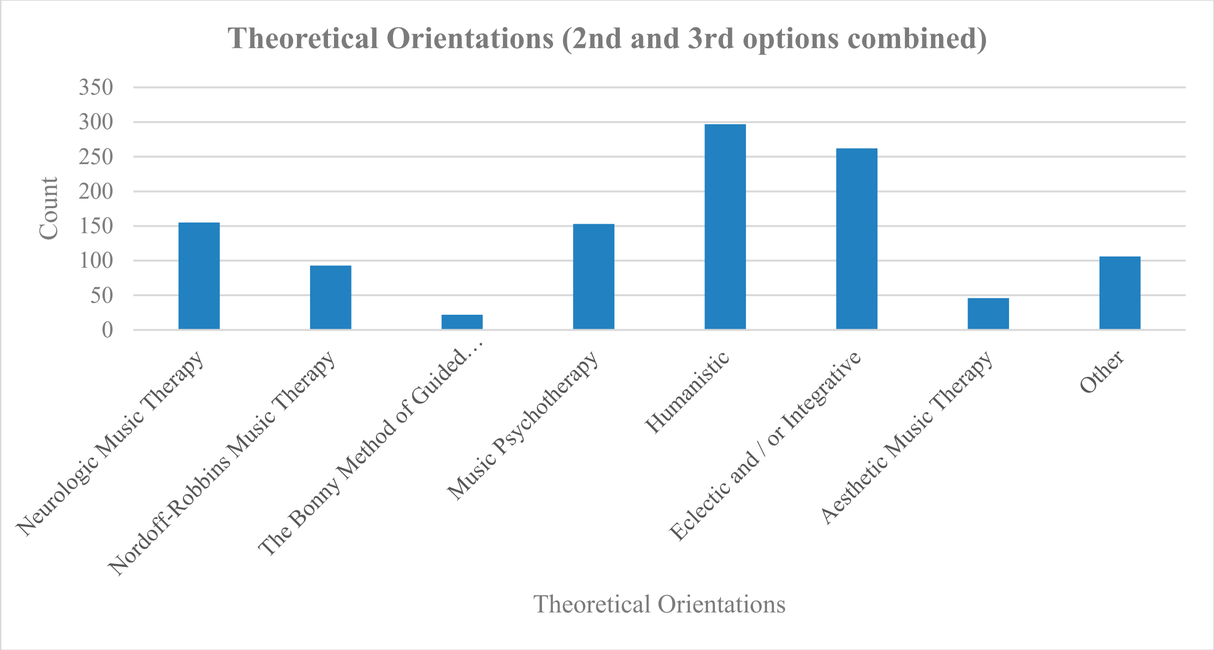

Theoretical Orientations (top 3 combined)

		Responses		Percent of Cases
		N	Percent	
TO123[a]	Neurologic Music Therapy	205	12.0%	35.8%
	Nordoff-Robbins Music Therapy	120	7.0%	21.0%
	The Bonny Method of Guided Imagery and Music	36	2.1%	6.3%
	Music Psychotherapy	200	11.7%	35.0%
	Humanistic	478	27.9%	83.6%
	Eclectic and / or Integrative	464	27.1%	81.1%
	Aesthetic Music Therapy	47	2.7%	8.2%
	Other	164	9.6%	28.7%
Total		1714	100.0%	299.7%

a. Group

8. What is your current primary work setting?

Work setting

	N	%
Currently Unemployed	9	1.6%
Private Practice	250	43.7%
Academia - Primary/Secondary Education	25	4.4%

Academia - Tertiary/Higher Education	30	5.2%
Healthcare/Medical facility	136	23.8%
Military or Veteran Medical Center	17	3.0%
Specialized Clinic	12	2.1%
Community Music/Arts Center	33	5.8%
Other	60	10.5%

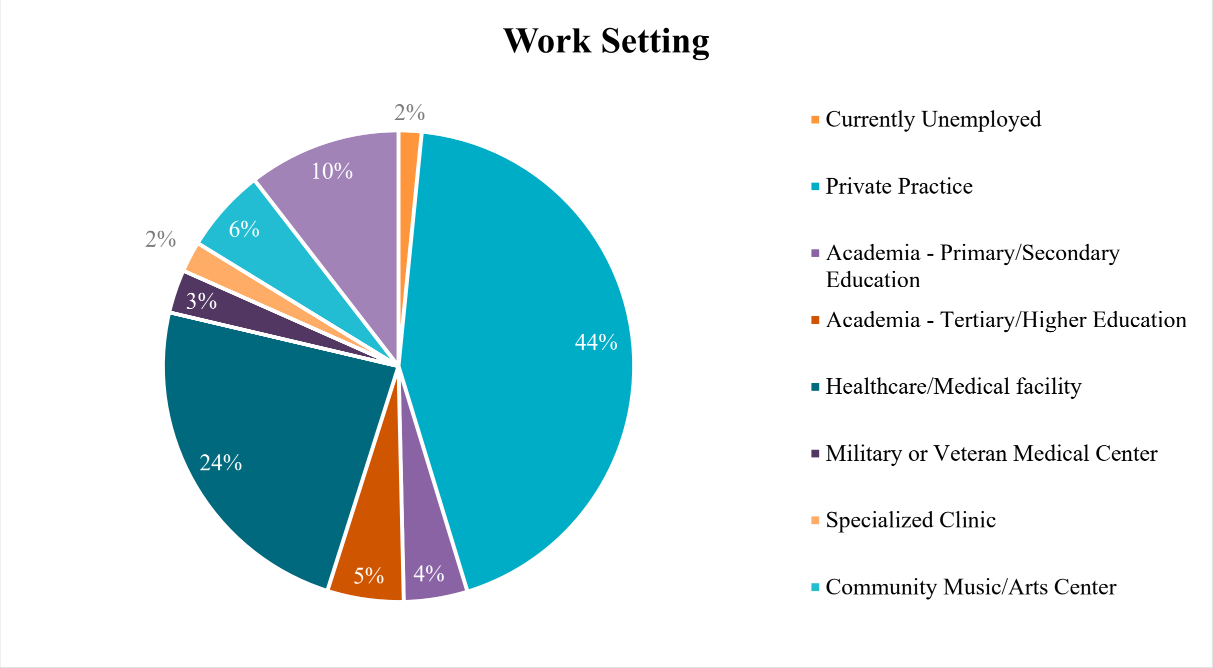

9. Prior to COVID-19, which clinical populations did you primarily serve?

Populations Served PRIOR to COVID-19 (up to 3)

		Responses		Percent of Cases
		N	Percent	
Populations PRIOR[a]	Children with developmental delays	281	20.1%	49.1%
	Persons with intellectual/multiples disabilities	255	18.3%	44.6%
	Children/youth at risk	72	5.2%	12.6%
	Adolescents	91	6.5%	15.9%
	Older adults	139	10.0%	24.3%
	Palliative care/End of life	86	6.2%	15.0%
	Dementia	101	7.2%	17.7%

	Medical/Oncology	61	4.4%	10.7%
	Premature infants	18	1.3%	3.1%
	Mental health	164	11.7%	28.7%
	Neurorehabilitation	81	5.8%	14.2%
	Other	47	3.4%	8.2%
Total		1396	100.0%	244.1%

a. Group

10. Since COVID-19, which clinical populations do you primarily serve?

Populations Served AFTER COVID-19 (up to 3)

		Responses		
		N	Percent	Percent of Cases
Populations AFTER[a]	Children with developmental delays	279	20.7%	48.8%
	Persons with intellectual/multiples disabilities	254	18.8%	44.4%
	Children/youth at risk	66	4.9%	11.5%
	Adolescents	110	8.2%	19.2%
	Older adults	143	10.6%	25.0%
	Palliative care/End of life	68	5.0%	11.9%
	Dementia	86	6.4%	15.0%
	Medical/Oncology	53	3.9%	9.3%
	Premature infants	15	1.1%	2.6%
	Mental health	180	13.4%	31.5%
	Neurorehabilitation	78	5.8%	13.6%
	Other	16	1.2%	2.8%
Total		1348	100.0%	235.7%

a. Group

11. How many clinical hours per week did you have prior to COVID-19?

Clinical hours prior to COVID-19

	N	%
Not applicable (instructor/administrator)	29	5.1%
1-9 hours per week	93	16.3%
10-19 hours per week	139	24.3%

20-29 hours per week	137	24.0%
30-35 hours per week	70	12.2%
36 hours or more per week	104	18.2%

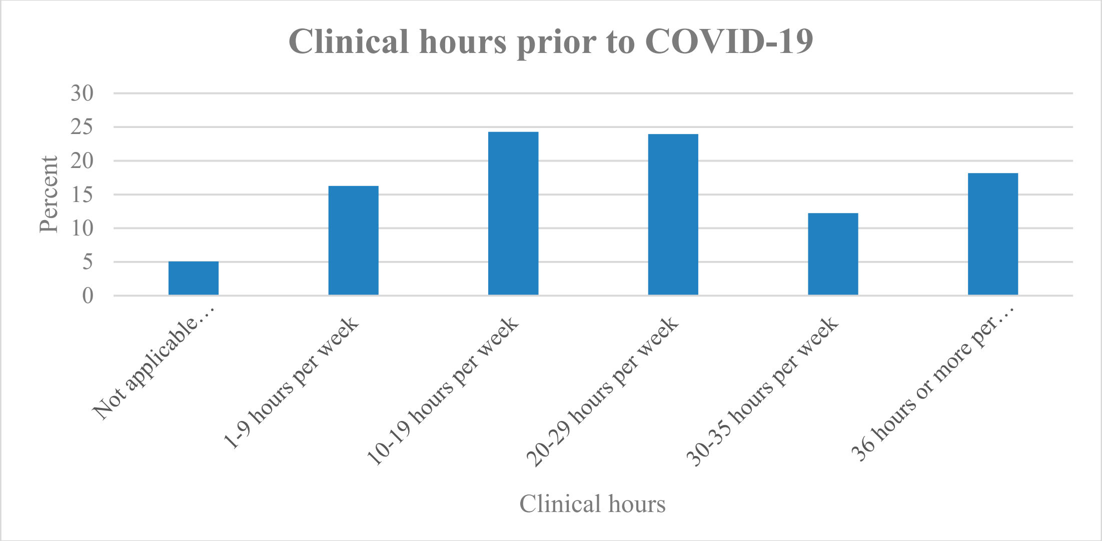

12. How many clinical hours per week have you had since COVID-19?

Clinical hours since COVID-19

	N	%
Not applicable (instructor/administrator)	14	2.4%
1-9 hours per week	140	24.5%
10-19 hours per week	130	22.7%
20-29 hours per week	131	22.9%
30-35 hours per week	62	10.8%
36 hours or more per week	95	16.6%

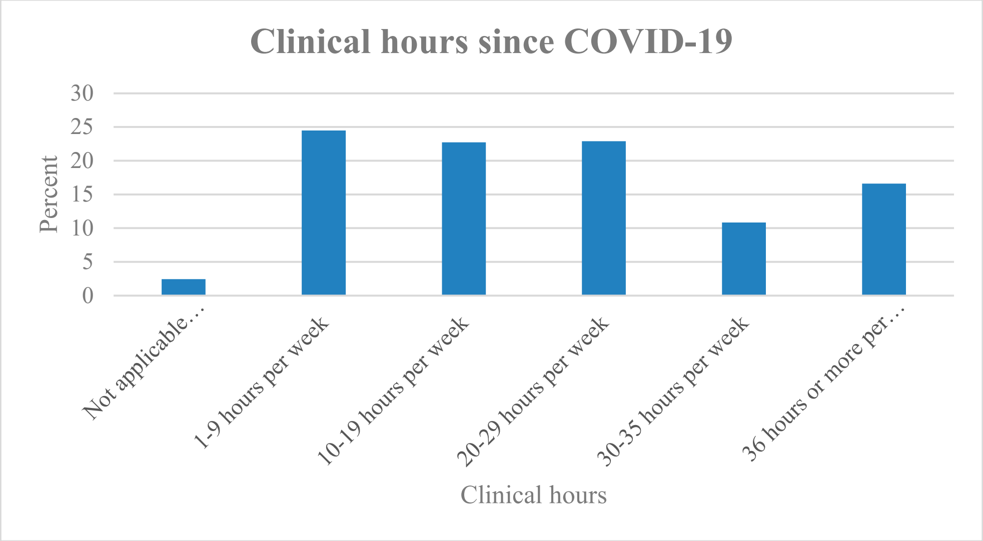

3. Telehealth Provision

13. What terminology do you most commonly use to describe your non-in-person MT services?

Terminology

	N	%
Online Music Therapy	115	20.1%
Remote Music Therapy	25	4.4%
Telehealth Music Therapy	262	45.8%
Virtual Music Therapy	148	25.9%
Distance Delivery of Music Therapy	2	0.3%
Other	20	3.5%

14. Have you been providing TMT prior to the pandemic? {For more than 5 sessions}

TMT prior to the pandemic

	N	%
Yes	73	12.8%
No	499	87.2%

15. What clinical needs do you address with TMT? {Select top three}

Clinical Needs Addressed with TMT (top 3)

		Responses		
		N	Percent	Percent of Cases
ClinicalNeedsTMT[a]	Pain	19	1.1%	3.3%
	Mood	234	13.6%	40.9%
	Anxiety	217	12.6%	37.9%
	Isolation	193	11.2%	33.7%
	Speech and Language / Communication	285	16.6%	49.8%
	Cognition	214	12.5%	37.4%
	Motor Function	107	6.2%	18.7%
	Spiritual Support	32	1.9%	5.6%
	Emotional Expression	363	21.2%	63.5%
	Other	52	3.0%	9.1%
Total		1716	100.0%	300.0%

a. Group

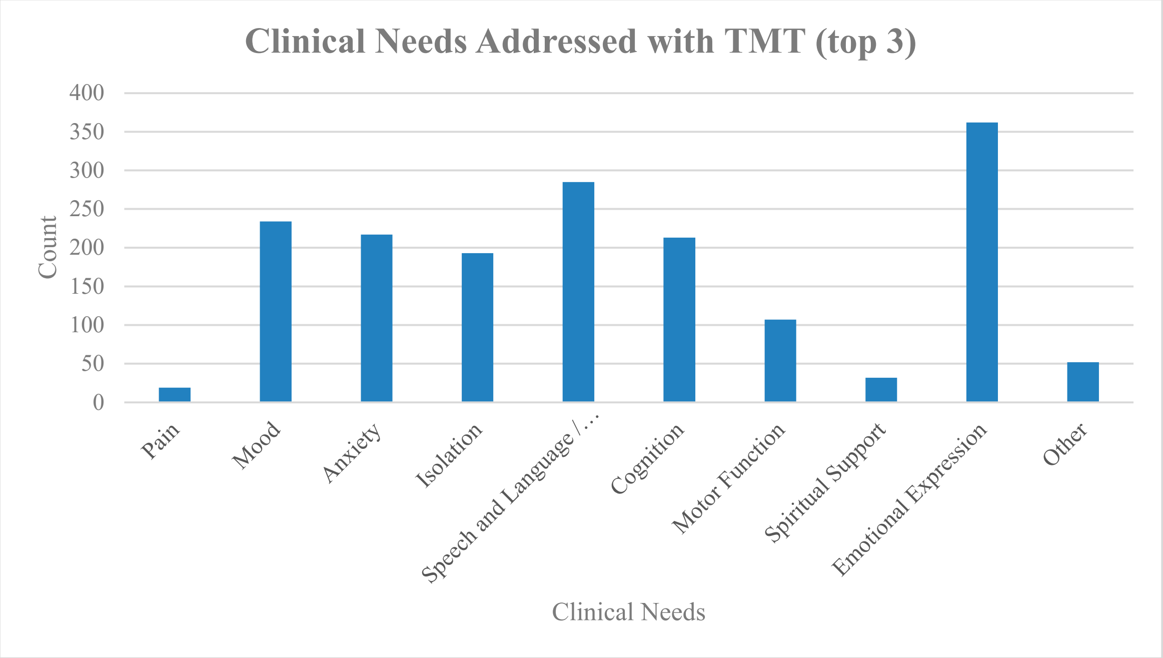

16. Have your client's goal areas changed due to the transition to telehealth compared to in-person sessions?

Change in client's goal areas (TMT vs. in-person)

		%
Not at all	86	15.0%
For some clients	241	42.1%
Unsure	79	13.8%
Likely	121	21.2%
Yes, definitely	45	7.9%

17. What are the most frequently used interventions during your TMT? {Select top two}

Most Frequently Used Interventions via Telehealth

		Responses		Percent of Cases
		N	Percent	
TMTInterventions2[a]	Music Listening	222	19.4%	38.8%
	Improvisation	108	9.4%	18.9%
	Songwriting	181	15.8%	31.6%
	Singing	310	27.1%	54.2%
	Mindfulness	84	7.3%	14.7%
	Movement to Music	178	15.6%	31.1%
	Other	61	5.3%	10.7%
Total		1144	100.0%	200.0%

a. Group

18. I have successfully used live music during:

Live music during in-person MT (success)

	N	%
Strongly disagree	2	0.3%
Disagree	2	0.3%
Neither agree nor disagree	7	1.2%
Agree	29	5.1%
Strongly agree	532	93.0%

Live music during TMT (success)

	N	%
Strongly disagree	10	1.7%

Disagree	29	5.1%
Neither agree nor disagree	59	10.3%
Agree	182	31.8%
Strongly agree	292	51.0%

19. I have successfully used <u>pre-recorded music</u> during:

Pre-recorded music during in-person MT (success)

	N	%
Strongly disagree	14	2.4%
Disagree	18	3.1%
Neither agree nor disagree	32	5.6%
Agree	85	14.9%
Strongly agree	423	74.0%

Pre-recorded music during TMT (success)

	N	%
Strongly disagree	20	3.5%
Disagree	27	4.7%
Neither agree nor disagree	52	9.1%
Agree	144	25.2%
Strongly agree	329	57.5%

20. What outcome measures do you use to assess progress in telehealth MT?

Outcome Measures in TMT

		Responses		
		N	Percent	Percent of Cases
OutcomeTMT[a]	Client Self-Report	347	26.3%	60.7%
	Family/Caregiver Report	318	24.1%	55.6%
	Observation	523	39.7%	91.4%
	Standardized Assessment	98	7.4%	17.1%
	Assessments performed by other healthcare workers	33	2.5%	5.8%
Total		1319	100.0%	230.6%

a. Group

21. In general, I am able to administer assessments over telehealth:

Ability to administer assessments over telehealth

	N	%
Strongly disagree	15	2.6%
Disagree	66	11.5%
Neither agree nor disagree	155	27.1%
Agree	254	44.4%
Strongly agree	82	14.3%

22. What online platform did you primarily use to administer TMT?

Online Platforms for TMT

		Responses		Percent of Cases
		N	Percent	
OnlinePlatforms[a]	Zoom	489	59.1%	85.5%
	Skype	40	4.8%	7.0%
	Teams	64	7.7%	11.2%
	Adobe Connect	5	0.6%	0.9%
	Facetime	69	8.3%	12.1%
	Google Duo	52	6.3%	9.1%
	Other	108	13.1%	18.9%
Total		827	100.0%	144.6%

a. Group

23. Did these platform(s) comply with legal or confidentiality requirements (e.g., HIPAA) in your country?

Platforms - legal/confidentiality requirements

	N	%
Yes	434	75.9%
No	14	2.4%
Not sure	124	21.7%

24. Did you take training, workshops, and/or courses to develop skills or increase proficiency of TMT service provision?

Additional Training

	N	%
Yes	287	50.2%
No	285	49.8%

4. Telehealth Perceptions

25. Do you anticipate continuing to provide TMT services post-pandemic?

Providing TMT post-pandemic

	N	%
Not at all	61	10.7%
For some clients	112	19.6%
Unsure	70	12.2%
Likely	104	18.2%
Yes, definitely	225	39.3%

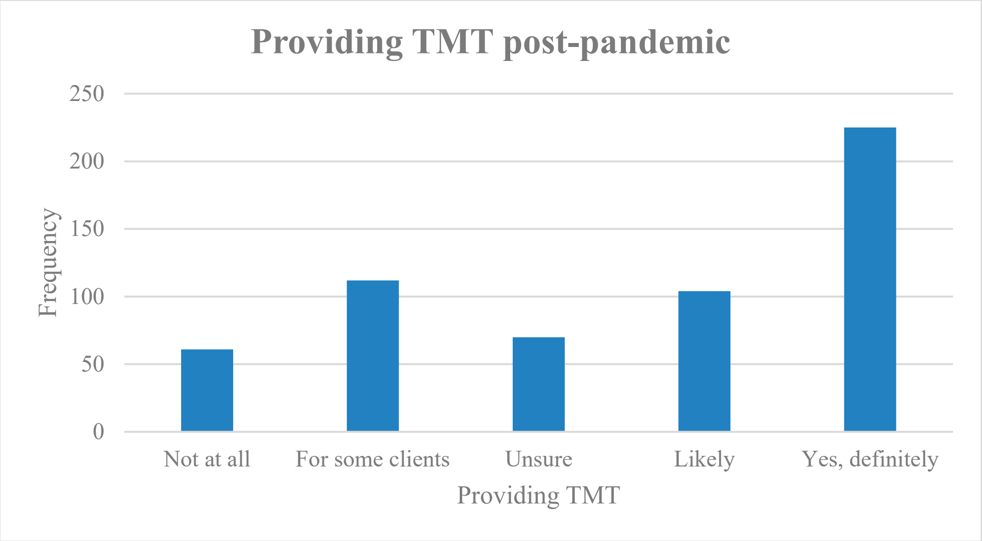

26. Do you anticipate that the frequency of your telehealth services will decline when restrictions for COVID-19 are removed completely?

Decline of TMT post COVID-19?

	N	%
Yes	440	76.9%
No	132	23.1%

26b. If yes, by what percentage (projected):

If yes, %

	N	%
By 10%	25	5.7
By 25%	69	15.8
By 50%	108	24.8
By 75%	234	53.7
Total	436	100

27. TMT has more benefits than drawbacks:

TMT has more benefits than drawbacks

	N	%
Strongly disagree	27	4.7%
Disagree	95	16.6%
Neither agree nor disagree	210	36.7%
Agree	152	26.6%
Strongly agree	88	15.4%

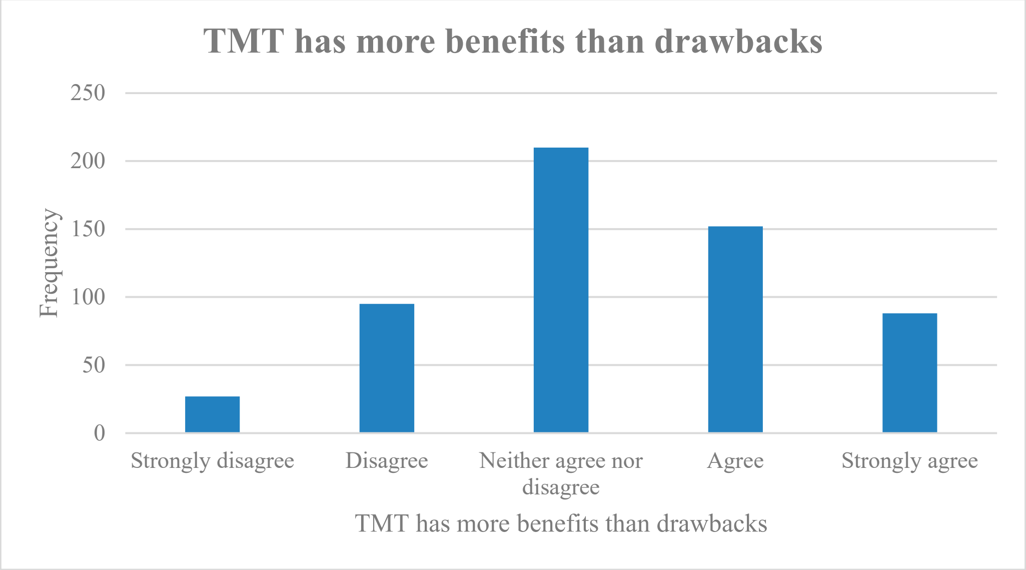

28. Caregiver involvement is beneficial for a telehealth model:

Caregiver involvement is beneficial for TMT

	N	%
Strongly disagree	6	1.0%
Disagree	13	2.3%
Neither agree nor disagree	128	22.4%
Agree	190	33.2%
Strongly agree	235	41.1%

29. Please list the main challenges for your clients in engaging in a telehealth MT session:

30. Please list the main challenges for yourself in engaging in a telehealth MT session:

31. Please list the main benefits for your clients in engaging in a telehealth MT session:

32. Please list the main benefits to you from engaging in a telehealth MT session:

33. If you are an MT supervisor, please describe challenges with:

a. Providing supervision in TMT

b. Supervisee skill acquisition in TMT
